# Investigations of the Binding of [Pt_2_(DTBPA)Cl_2_](II) and [Pt_2_(TPXA)Cl_2_](II) to DNA via Various Cross-Linking Modes

**DOI:** 10.3390/ijms141019556

**Published:** 2013-09-26

**Authors:** Hongwei Yue, Bo Yang, Yan Wang, Guangju Chen

**Affiliations:** College of Chemistry, Beijing Normal University, Beijing 100875, China; E-Mails: yuehongwei0813@126.com (H.Y.); yangb@bnu.edu.cn (B.Y.)

**Keywords:** molecular dynamics simulations, platinum-DNA adduct, [Pt_2_(DTBPA)Cl_2_](II), [Pt_2_(TPXA)Cl_2_](II), conformational distortion

## Abstract

We have constructed models for a series of platinum-DNA adducts that represent the binding of two agents, [Pt_2_(DTBPA)Cl_2_](II) and [Pt_2_(TPXA)Cl_2_](II), to DNA via inter- and intra-strand cross-linking, and carried out molecular dynamics simulations and DNA conformational dynamics calculations. The effects of *trans*- and *cis*-configurations of the centers of these di-nuclear platinum agents, and of different bridging linkers, have been investigated on the conformational distortions of platinum-DNA adducts formed via inter- and intra-strand cross-links. The results demonstrate that the DNA conformational distortions for the various platinum-DNA adducts with differing cross-linking modes are greatly influenced by the difference between the platinum-platinum distance for the platinum agent and the platinum-bound N7–N7 distance for the DNA molecule, and by the flexibility of the bridging linkers in the platinum agent. However, the effects of *trans*/*cis*-configurations of the platinum-centers on the DNA conformational distortions in the platinum-DNA adducts depend on the inter- and intra-strand cross-linking modes. In addition, we discuss the relevance of DNA base motions, including opening, shift and roll, to the changes in the parameters of the DNA major and minor grooves caused by binding of the platinum agent.

## 1. Introduction

Platinum-based drugs, such as cisplatin, carboplatin and oxaliplatin, are agents that have been used effectively to treat various types of human cancer, including genitourinary, colorectal and non-small cell lung cancers [1–5]. Various investigations have revealed that DNA is most likely the biological target associated with the antitumor activity of these drugs [6,7].

The platinum at the center of these agents binds to nucleophilic sites of DNA (the N7 atoms of guanosine [G] and adenosine [A] bases), and these interactions result in distortions of the DNA conformation that are critical to the antitumor effects [8–10]. However, the use of these platinum-based agents has often been limited by the development of tumor resistance [11–14]. Therefore, considerable effort has been made to develop new generations of platinum agents that do not share these limitations [15–17]. Polynuclear platinum agents represent a novel class of antitumor metallodrugs, in which two or three platinum centers are bridged by polyamines of variable length. These agents bind to DNA by forming various long-range intra-strand and inter-strand cross-links [18–20]. This unique DNA-binding feature is believed to be responsible for distortion of the DNA conformation, enhanced cytotoxicity, and effective prevention of resistance to the antitumor drug [21–23].

**Chart 1:**
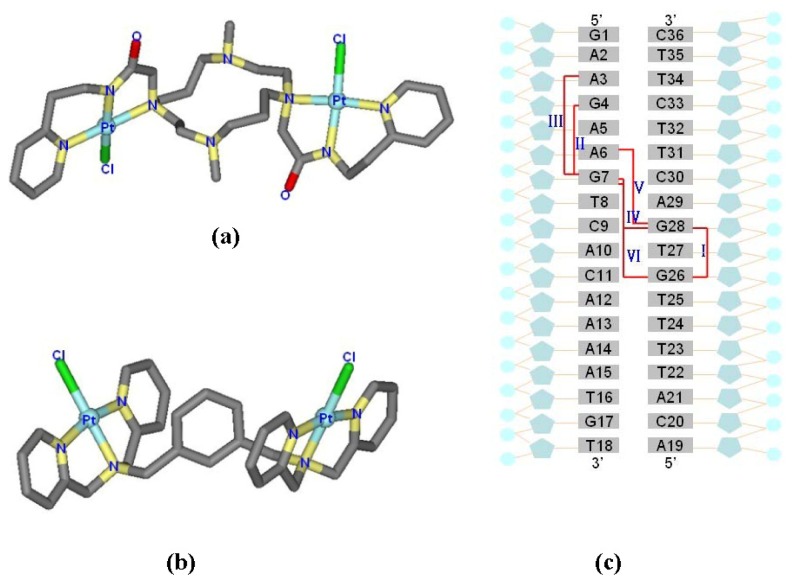
X-ray structures for (**a**) [Pt_2_(DTBPA)Cl_2_](II); and (**b**) [Pt_2_(TPXA)Cl_2_](II); (**c**) the 18-mer duplex DNA sequence and the six platinum-DNA cross-linking modes for [Pt_2_(DTBPA)Cl_2_](II) studied in this work, along with their assigned names. Three types of intra-strand cross-links (I: 26.28DTBPA-DNA; II: 4.7DTBPA-DNA; and III: 3.7DTBPA-DNA), and three types of inter-strand cross-links (IV: 7.28DTBPA-DNA; V: 6.28DTBPA-DNA; and VI: 7.26DTBPA-DNA) were studied. Similar nomenclature was used for [Pt_2_(TPXA)Cl_2_](II).

The di-nuclear platinum(II) agents, [Pt_2_(DTBPA)Cl_2_](II) (where DTBPA = 2,20-(4,11-dimethyl- 1,4,8,11-tetraazacyclotetradecane-1,8-diyl)bis (*N*-(2-(pyridin-2-yl)ethyl)acetamide)) and [Pt_2_(TPXA)Cl_2_](II) (where TPXA = *N*,*N*,*N*′,*N*′-tetra(2-pyridylmethyl)-m-xylylene diamine), are two examples of polynuclear platinum drugs that are anticancer therapeutic agents [24,25]. In the [Pt_2_(DTBPA)Cl_2_](II) agent, two platinum centers are bridged by a cyclam ring, and each platinum center is coordinated by one chloride and three nitrogens in, respectively, the cyclam ring, acetamide and pyridyl groups (see Chart 1) [24]. In the [Pt_2_(TPXA)Cl_2_](II) agent, two platinum centers are bridged by a benzene linker, and each platinum center is coordinated by one chloride and three nitrogens in the bis(2-pyridylmethyl)amine (BPA) moiety (see Chart 1) [25]. Previous experiments have demonstrated that both agents exhibit significantly enhanced cytotoxicity against cancers compared with cisplatin [19–22]. Whereas cisplatin binds to DNA by forming short-range cross-links [26–28], the two di-nuclear platinum(II) agents, which have a cyclam ring or benzene group as a bridged linker, can bind to DNA by forming long-range inter-/intra-strand cross-links [24,25]. Moreover, the features of the multi-ring ligands in these two di-nuclear platinum(II) agents may facilitate platinum-DNA interactions through pre-associative electrostatic and H-bonding interactions [29–32]. Taking into account the platinum-platinum distances of 9.1–9.7 Å in the [Pt_2_(DTBPA)Cl_2_](II) and [Pt_2_(TPXA)Cl_2_](II) agents, and the distance of about 3.4 Å between two neighboring base pairs in B-DNA [24,25,33,34], it would be predicted that in the platinum-DNA adducts, the two platinum centers of [Pt_2_(DTBPA)Cl_2_](II) or [Pt_2_(TPXA)Cl_2_](II) would be capable of spanning 3–5 base pairs. Theoretically, Mori *et al*., using *ab initio* fragment molecular orbital-based molecular dynamics (FMO-MD) simulations, presented that the Pt–Cl bond fluctuation and charge distribution for *cis*- and *trans*-platinum are important for activating their anticancer functions [35,36]. However, few theoretical studies devoted to the properties of the [Pt_2_(DTBPA)Cl_2_](II) and [Pt_2_(TPXA)Cl_2_](II) agents binding to DNA molecules.

The [Pt_2_(DTBPA)Cl_2_](II) agent, with two *trans*-platinum centers and a large 14-membered ring linker (cyclam), and the [Pt_2_(TPXA)Cl_2_](II) agent, with two *cis*-platinum centers and a small 6-membered ring linker (benzene), exhibit distinct structural features that may influence their DNA binding modes and DNA distortion properties. However, how the structures of these two platinum agents affect their interaction and binding with DNA is currently not fully understood at the molecular level. In the present work, we have used molecular dynamics simulations and DNA conformational dynamics analyses to study variations in the conformations of the [Pt_2_(DTBPA)Cl_2_](II)-DNA and [Pt_2_(TPXA)Cl_2_](II)-DNA adducts with different cross-linking modes. Six different DNA-adduct models for each platinum agent, with inter-/intra-strand cross-linking modes of 1–3, 1–4 and 1–5 base intervals, were investigated to examine the properties of the DNA distortions induced by the two platinum agents.

## 2. Results

**Figure 1:**
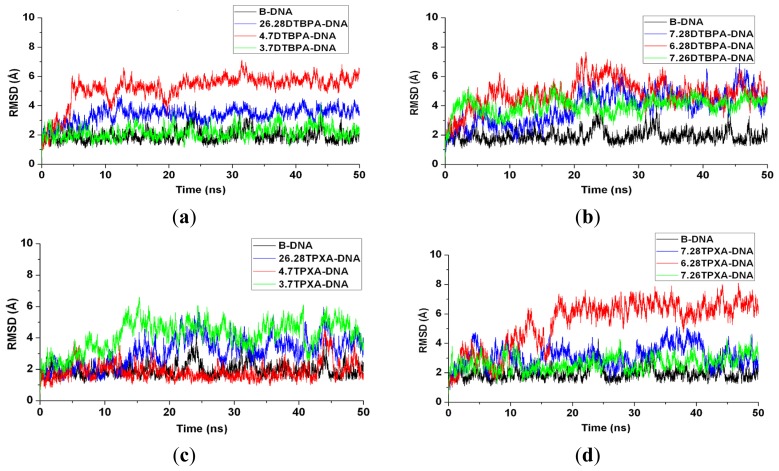
Root-mean-square deviation (RMSD) values of all the backbone atoms with respect to the corresponding starting structures for: (**a**) simulations of B-DNA (black), 26.28DTBPA-DNA (blue), 4.7DTBPA-DNA (red) and 3.7DTBPA-DNA (green); (**b**) simulations of B-DNA (black), 7.28DTBPA-DNA (blue), 6.28DTBPA-DNA (red) and 7.26DTBPA-DNA (green); (**c**) simulations of B-DNA (black), 26.28TPXA-DNA (blue), 4.7TPXA-DNA (red) and 3.7TPXA-DNA (green); (**d**) simulations of B-DNA (black), 7.28TPXA-DNA (blue), 6.28TPXA-DNA (red) and 7.26TPXA-DNA (green).

**Figure 2:**
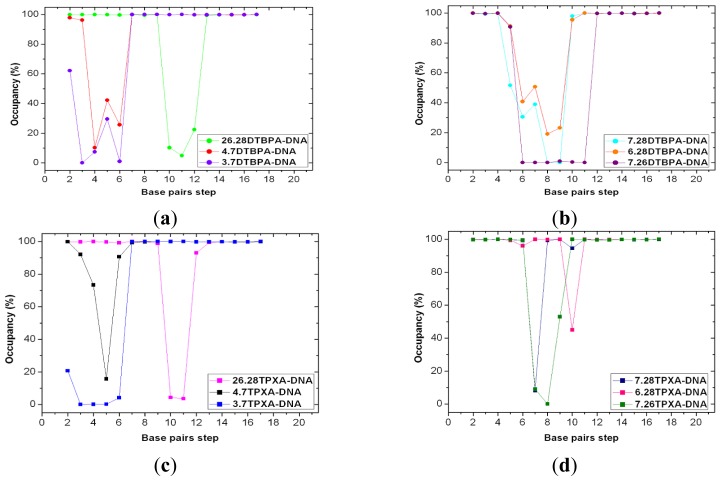
Hydrogen bond occupancies of the base pairs and the base-pair steps for the various platinum-DNA adducts (shown as circles for [Pt_2_(DTBPA)Cl_2_(II)] and squares for [Pt_2_(TPXA)Cl_2_(II)]). (**a**) 26.28DTBPA-DNA (green), 4.7DTBPA-DNA (red) and 3.7DTBPA-DNA (violet); (**b**) 7.28DTBPA-DNA (cyan), 6.28DTBPA-DNA (orange) and 7.26DTBPA-DNA (purple); (**c**) 26.28TPXA-DNA (magenta), 4.7TPXA-DNA (black) and 3.7TPXA-DNA (blue); (**d**) 7.28TPXA-DNA (navy), 6.28TPXA-DNA (pink) and 7.26TPXA-DNA (olive).

**Figure 3:**
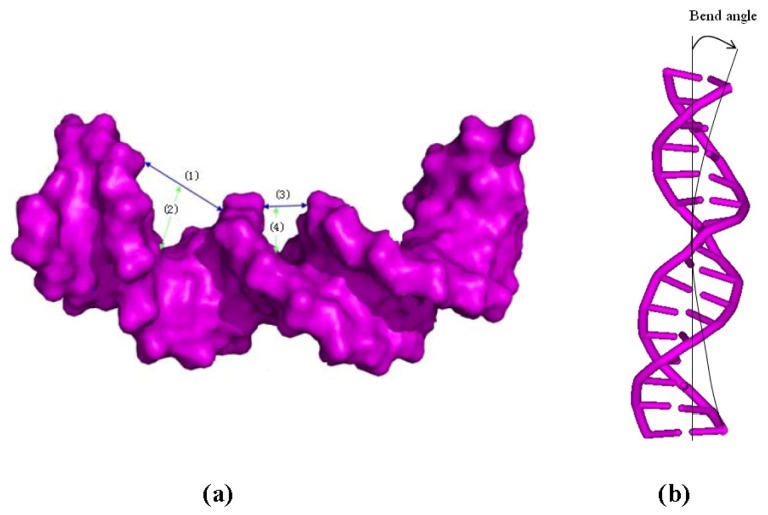
(**a**) The width and depth of the major groove of the time-averaged DNA conformation are shown as blue line (**1**) and green line (**2**), respectively, and the width and depth of the minor groove are shown as blue line (**3**) and green line (**4**), respectively; (**b**) The bend angles of the time-averaged DNA conformation.

**Figure A1:**
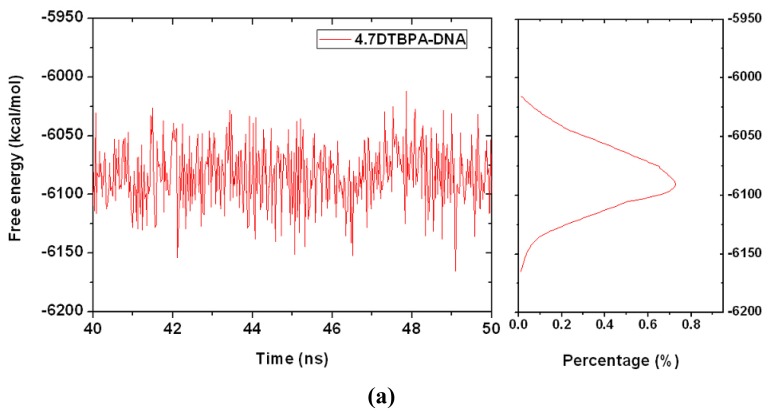

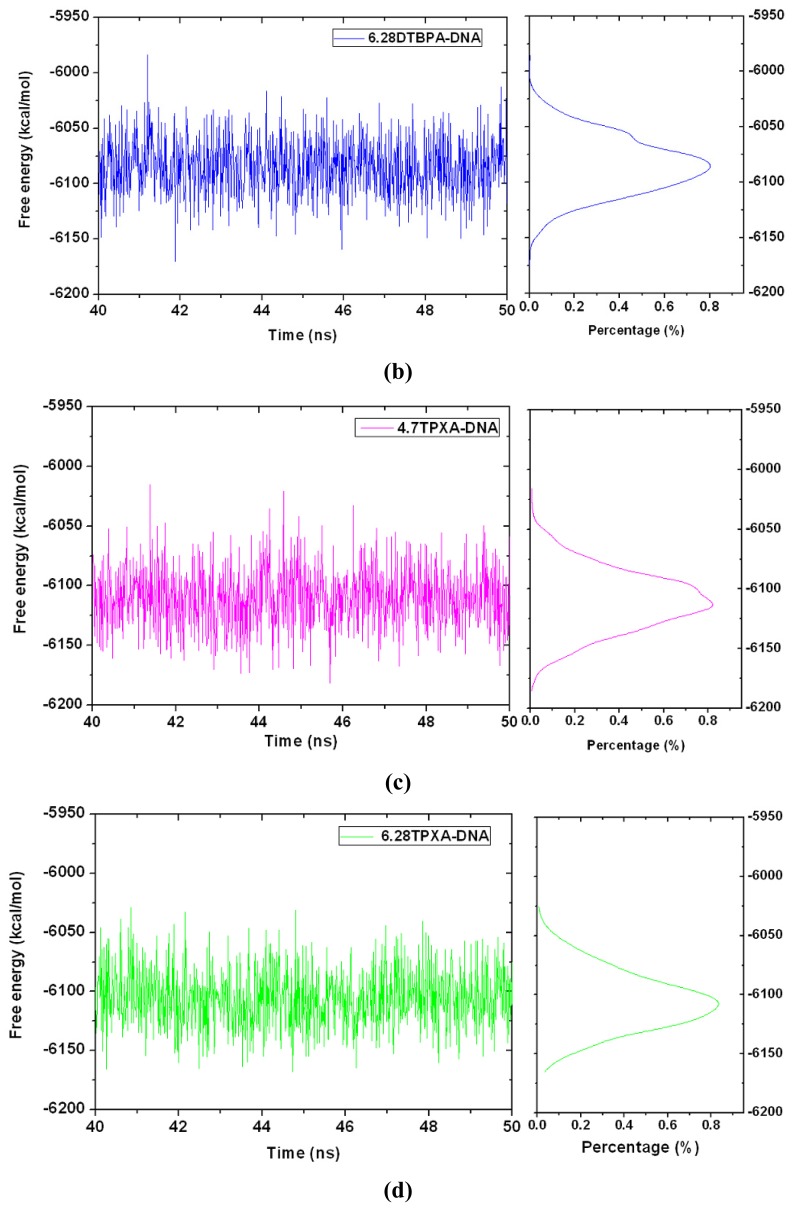
Conformational stability free energy (kcal·mol^−1^) and integrated distributions for (**a**) 4.7DTBPA-DNA (red); (**b**) 6.28DTBPA-DNA (blue), (**c**) 4.7TPXA-DNA (magenta) and (**d**) 6.28TPXA-DNA (green).

The 12 platinum-DNA adduct models (six [Pt_2_(DTBPA)Cl_2_](II)+DNA adducts: 26.28DTBPA-DNA, 4.7DTBPA-DNA, 3.7DTBPA-DNA, 7.28DTBPA-DNA, 6.28DTBPA-DNA and 7.26DTBPA-DNA; and six [Pt_2_(TPXA)Cl_2_](II) + DNA adducts: 26.28TPXA-DNA, 4.7TPXA-DNA, 3.7TPXA-DNA, 7.28TPXA-DNA, 6.28TPXA-DNA and 7.26TPXA-DNA) were studied by performing 50-ns unrestrained molecular dynamics simulations. The root-mean-square deviation (RMSD) values for all the backbone atoms, referenced to the corresponding starting structures, over 12 trajectories for the various intra-/inter-strand cross-linking models of the platinum-DNA adduct, were examined to determine if each system had attained equilibrium. A small RMSD value for a simulation is often considered to indicate that the system is in a stable state, whereas large RMSD values suggest the occurrence of large conformational changes. Plots of the RMSDs of the 12 platinum-DNA system simulations over time are shown in Figure 1. The calculated conformational free energies of the platinum-DNA systems as a function of time, during the last 10 ns of the simulation, were analyzed [5,37]. The corresponding results for the 4.7DTBPXA-DNA, 6.28DTBPA-DNA, 4.7TPXA-DNA and 6.28TPXA-DNA models are shown in Figure A1 (a, b, c and d, respectively). It may be seen from Figures 1 and A1 that the platinum-DNA adduct systems reached equilibrium after 10 ns, and that their energies were found to be stable during the remainder of each simulation. Therefore, the trajectory analysis for the 12 systems extracted the equilibrated conformations between 10 ns and 50 ns of the simulation time, recording 20,000 snapshots at 2-ps time intervals for each trajectory. The analyses of the hydrogen bonds of the DNA base pairs and the DNA-groove parameters around the binding sites of the DNA were performed using the time-averaged structures for these models, and are shown in Table 1, Figures 2 and 3.

### 2.1. Conformational Characteristics and Flexibility of [Pt_2_(DTBPA)Cl_2_](II) and [Pt_2_(TPXA)Cl_2_](II)

**Figure 4:**
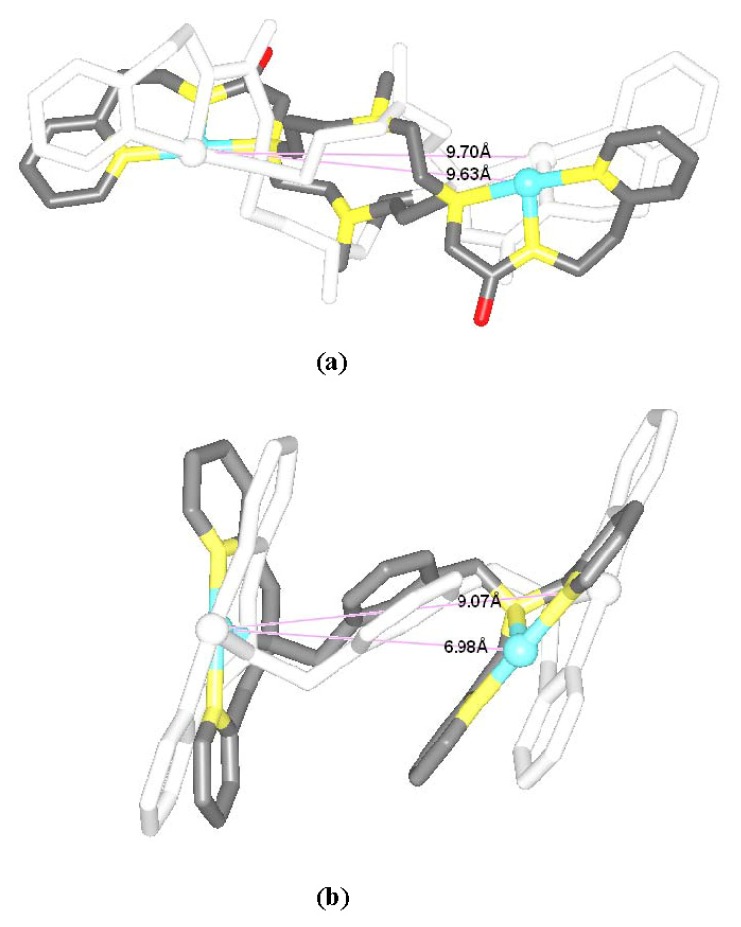
Overlap conformations between the crystal structures (white) and the averaged simulated structures of (**a**) [Pt_2_(DTBPA)Cl_2_](II) (gray); and (**b**) [Pt_2_(TPXA)Cl_2_](II) (gray). The platinum atoms are shown as white in the crystal structures, and light cyan in the averaged structures.

The [Pt_2_(DTBPA)Cl_2_](II) agent consists of a large cyclam ring linker and two *trans*-platinum(II) centers, each of which is coordinated by one chloride and one nitrogen each from the cyclam ring acetamide and pyridyl groups. The [Pt_2_(TPXA)Cl_2_](II) agent consists of a small benzene ring linker and two *cis*-platinum(II) centers, each of which is coordinated in a square-planar geometry by one chloride and three nitrogen atoms from each bis(2-pyridylmethyl)amine (BPA) moiety. The conformational characteristics of the DNA binding processes for these two platinum(II) antitumor agents are expected to differ, due to the different ligands and linkers of the platinum(II) centers [24,25]. In order to compare the conformational flexibility of [Pt_2_(DTBPA)Cl_2_](II) and [Pt_2_(TPXA)Cl_2_](II), the average platinum-platinum distances of these platinum-DNA adducts were evaluated before and after simulation, using the conformation overlap method. Figure 4 shows the full-overlap conformations of [Pt_2_(DTBPA)Cl_2_](II) and [Pt_2_(TPXA)Cl_2_](II) between the crystal structures and averaged structures during the 50-ns simulations. It may be seen from Figure 4 that the average platinum-platinum distance values for [Pt_2_(DTBPA)Cl_2_](II) and [Pt_2_(TPXA)Cl_2_](II) change, during simulation, from 9.70 Å and 9.07 Å in the crystal structures to 9.63 Å and 6.98 Å in the platinum-DNA adducts. Such small rangeability of the platinum-platinum distance for [Pt_2_(DTBPA)Cl_2_](II), which has a large-sized linker, during the simulations demonstrates that it has a more rigid conformation than [Pt_2_(TPXA)Cl_2_](II), which has a smaller-sized linker.

### 2.2. Relationship between the Platinum–Platinum Distances in the Platinum(II) Agents and the N7–N7 Distances in the Pt_2_(DTBPA)-DNA and Pt_2_(TPXA)-DNA Adducts

**Figure 5:**
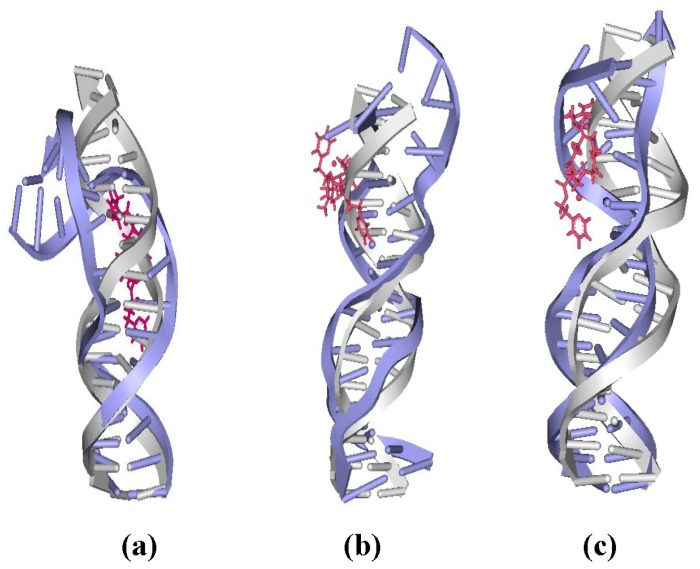
The averaged centroid structures of the platinum-DNA adduct and an undamaged B-DNA for: (**a**) 26.28DTBPA-DNA; (**b**) 4.7DTBPA-DNA; and (**c**) 3.7DTBPA-DNA. [Pt_2_(DTBPA)Cl_2_](II) is shown as pink, bound-DNA as blue, and B-DNA as gray.

To address the effects of the platinum–platinum distance and platinum-bound N7–N7 distance on the extent of the distortion of the DNA conformation in the platinum-DNA adduct, the overlaps of the averaged structures for intra-1,3-GG cross-linking 26.28DTBPA-DNA, intra-1,4-GG cross-linking 4.7DTBPA-DNA and intra-1,5-GG cross-linking 3.7DTBPA-DNA were compared with a bare DNA molecule (Figure 5). The platinum-bound N7–N7 distance in the three models was used as a test parameter for the degree of the DNA conformational distortion. The platinum-bound N7–N7 distances in DNA and Pt–Pt distances in the platinum agents for the three models and for the B-DNA molecule are shown in Table 2. Of the three models, the platinum–platinum distance in the [Pt_2_(DTBPA)Cl_2_](II) agent most closely corresponded to the platinum-bound N7–N7 distance in the intra-1,4-GG cross-linking 4.7DTBPA-DNA. It may be seen from Table 2 that the difference in the N7–N7 distance between the B-DNA molecule and the averaged platinum-bound adduct was 2.22 Å for the intra-1,3-GG cross-linking 26.28DTBPA-DNA, and 3.76 Å for the intra-1,5-GG cross-linking 3.7DTBPA-DNA adducts, but only 0.38 Å for the intra-1,4-GG cross-linking 4.7DTBPA-DNA. Moreover, as shown in Figure 2a, the sum of the percentages of the hydrogen bonds destroyed at the bases, in comparison with the B-DNA molecule, was 262.55% for 26.28DTBPA-DNA (A10, 89.79%; C11, 95.13%; and A12, 77.63%) and 404.57% for 3.7DTBPA-DNA (A2, 37.87%; A3, 100%; G4, 92.66%; A5, 75%; and A6, 99.04%), but only 202.05% for 4.7DTBPA-DNA (G4, 69.88%; A5, 57.79%; and A6, 74.38%). The differences in the bend angles of the DNA molecule toward the minor groove between the B-DNA molecule and each adduct were 20.46° for 26.28DTBPA-DNA, 33.60° for 3.7DTBPA-DNA, and only 15.68° for 4.7DTBPA-DNA (see Table 1 and Figure 3). As would be expected, Figure 5 indicates visually that the distortion of the average DNA conformation, compared with the B-DNA molecule, was also larger for both 26.28DTBPA-DNA and 3.7DTBPA-DNA models than for 4.7DTBPA-DNA model. Therefore, large differences between the platinum–platinum distance in the platinum agent and the platinum-bound N7–N7 distance in the DNA molecule can cause substantial distortions of the DNA conformation during the process of platinum-DNA adduct formation. Similar results were observed for the [Pt_2_(TPXA)Cl_2_](II) agent and the inter-strand cross-linking models, except for the fact that the effect on the conformationally-rigid [Pt_2_(DTBPA)Cl_2_](II) agent was more obvious than that on [Pt_2_(TPXA)Cl_2_](II), which has a more flexible conformation.

### 2.3. Relationship between the Flexibilities of the [Pt_2_(DTBPA)Cl_2_](II) and [Pt_2_(TPXA)Cl_2_](II) Agents and Their DNA Binding Interactions

**Figure 6:**
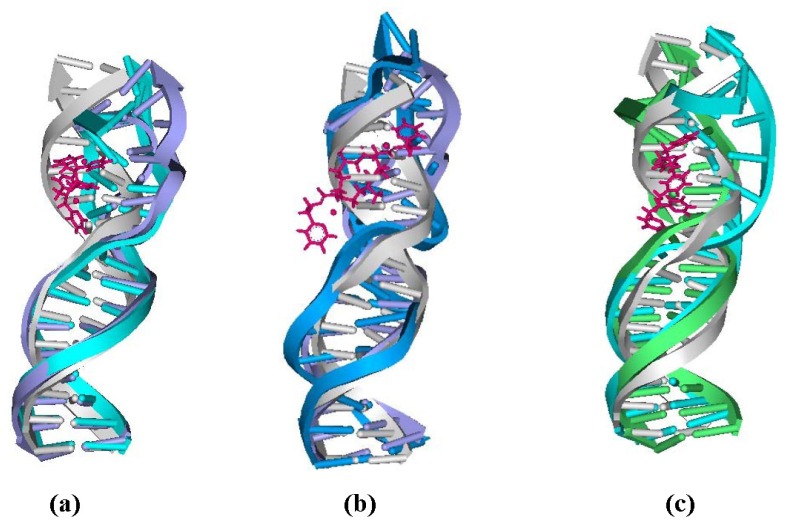
Overlap of the averaged structures for B-DNA (gray), [Pt_2_(DTBPA)Cl_2_](II) and [Pt_2_(TPXA)Cl_2_](II) (pink), and: (**a**) 4.7DTBPA-DNA (blue) and 4.7TPXA-DNA (cyan); (**b**) 4.7DTBPA-DNA (blue) and 6.28DTBPA-DNA (royal); and (**c**) 4.7TPXA-DNA (cyan) and 6.28TPXA-DNA (green).

Based on the fact that the [Pt_2_(DTBPA)Cl_2_](II) agent has more rigidity than the [Pt_2_(TPXA)Cl_2_](II) agent, a comparison was made of the DNA conformational distortions caused by the rigid [Pt_2_(DTBPA)Cl_2_](II) agent and the flexible [Pt_2_(TPXA)Cl_2_](II) agent, using the 4.7DTBPA-DNA and 4.7TPXA-DNA adducts. Figure 6a shows the overlaps of the averaged structures for these two models, which indicate that the distortion of the DNA conformation for the 4.7DTBPA-DNA model was larger than that for the 4.7TPXA-DNA model. The sum of the percentages of the hydrogen bonds destroyed at bases G4, A5 and A6, in comparison with the B-DNA molecule, was 202.05% for the 4.7DTBPA-DNA adduct, and only 120.28% for the 4.7TPXA-DNA adduct (see Figure 2a,c). The average deviation of the major groove width, depth and bend angle were also much larger for the 4.7DTBPA-DNA adduct (16.17% [from 11.81 Å to 13.72 Å], 44.86% [from 5.84 Å to 3.22 Å] and 104.39% [from 15.02° to 30.70°], respectively) than for the 4.7TPXA-DNA adduct (6.09% [from 11.81 Å to 12.53 Å], 14.21% [from 5.84 Å to 5.01 Å] and 74.30% [from 15.02° to 26.18°], respectively) (see Table 1 and Figure 3). Similar results for the other inter-/intra-strand cross-linking models were observed. Consequentially, the more rigid platinum adducts may lead to obvious distortion of the DNA backbone.

### 2.4. Relationship between the trans-[Pt_2_(DTBPA)Cl_2_](II)/cis-[Pt_2_(TPXA)Cl_2_](II) Configurations and the DNA Inter-/Intra-Strand Cross-Linking Modes

To investigate the effects of *trans*-[Pt_2_(DTBPA)Cl_2_](II) and *cis*-[Pt_2_(TPXA)Cl_2_](II) conformations on the distortion of the DNA conformation in the intra-/inter-strand cross-linking modes of the platinum-DNA adducts, comparisons were made of the DNA conformational distortions between the B-DNA molecule and each of the intra-1,4-GG cross-linking modes of the 4.7DTBPA-DNA and 4.7TPXA-DNA adducts, and the inter-1,4-GG cross-linking modes of the 6.28DTBPA-DNA and 6.28TPXA-DNA adducts. Figures 6b,c show the overlaps of the averaged structures for these four adducts, indicating that for the *trans*-type platinum agent, the distortion of the DNA conformation (in comparison with the B-DNA molecule) was larger for the inter-strand cross-linking 6.28DTBPA-DNA model than for the intra-strand cross-linking 4.7DTBPA-DNA model; in contrast, for the *cis*-type platinum agent, the DNA conformational distortion was larger for the intra-strand cross-linking 4.7TPXA-DNA model than for the inter-strand cross-linking 6.28TPXA-DNA model. The sum of the hydrogen bonds destroyed at the bases for *trans*-[Pt_2_(DTBPA)Cl_2_](II) was 282.06% for the inter-strand cross-linking 6.28DTBPA-DNA adduct (A5, 10.91%; A6, 58.14%; G7, 48.16%; T8, 81.05%; C9, 78.43%; and A10, 5.37%), much larger than the value of 202.05% for the intra-strand cross-linking 4.7DTBPA-DNA adduct (at the G4, A5 and A6 bases) (see Figure 2a,b). In contrast, for the *cis*-[Pt_2_(TPXA)Cl_2_](II) agent, the sum of the hydrogen bonds destroyed at bases G4, A5 and A6 was 120.28% for the intra-strand cross-linking 4.7TPXA-DNA adduct, much larger than the value of 55.04% at only one base (A10) for the inter-strand cross-linking 6.28TPXA-DNA adduct (see Figure 2c,d). Similarly, for the *trans*-[Pt_2_(DTBPA)Cl_2_](II) agent, the average deviations of the major groove width, depth and bend angle, in comparison with the B-DNA molecule, were larger for the inter-strand cross-linking 6.28DTBPA-DNA adduct (33.44% [from 11.81 Å to 15.76 Å], 57.87% [from 5.84 Å to 2.46 Å] and 232.29% [from 15.02° to 49.91°], respectively) than for the intra-strand cross-linking 4.7DTBPA-DNA adduct (16.17% [from 11.81 Å to 13.72 Å], 44.86% [from 5.84 Å to 3.22 Å] and 104.39% [from 15.02° to 30.70°], respectively). In contrast, for the *cis*-[Pt_2_(TPXA)Cl_2_](II) agent, the corresponding values were larger for the intra-strand cross-linking 4.7TPXA-DNA adduct (6.09% [from 11.81 Å to 12.53 Å], 14.21% [from 5.84 Å to 5.01 Å] and 74.30% [from 15.02° to 26.18°], respectively) than for the inter-strand cross-linking 6.28TPXA-DNA adduct (1.44% [from 11.81 Å to 11.98 Å], 1.88% [from 5.84 Å to 5.73 Å] and 80.76% [from 15.02° to 27.15°], respectively) (see Table 1 and Figure 3). Similar results were observed for other inter-/intra-strand cross-linking models of these two platinum agents. Taken together, these data suggest that the *trans*-type platinum agents facilitate greater DNA conformational distortion through an inter-strand rather than an intra-strand cross-linking mode, whereas the *cis*-type platinum agents create greater DNA conformational distortion through an intra-strand rather than an inter-strand cross-linking mode [38–40]. Furthermore, platinum agents with *trans*-type DNA cross-links have been shown to have high antitumor activity in cytotoxicity experiments [41].

### 2.5. Configuration Characteristics of Two Additional Platinum Agents, trans-[Pt_2_(DPZM)Cl_2_](II) and cis-[Pt_2_(DPZMCH2)Cl_2_](II), Binding to DNA

**Figure A2:**
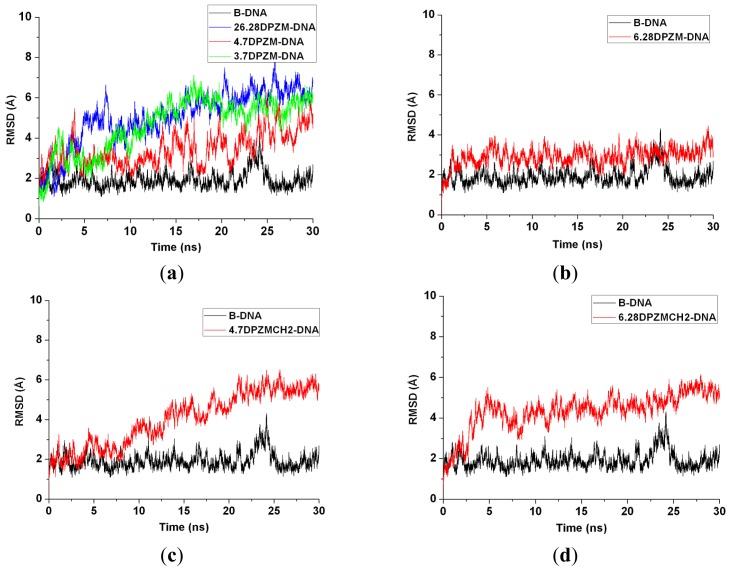
RMSD values of all the backbone atoms with respect to the corresponding starting structures for: (**a**) simulations of B-DNA (black), 26.28DPZM-DNA (blue), 4.7DPZM-DNA (red) and 3.7DPZM-DNA (green); (**b**) simulations of B-DNA (black) and 6.28DPZM-DNA (red); (**c**) simulations of B-DNA (black) and 4.7DPZMCH2-DNA (red); (**d**) simulations of B-DNA (black) and 6.28DPZMCH2-DNA (red).

**Figure A3:**
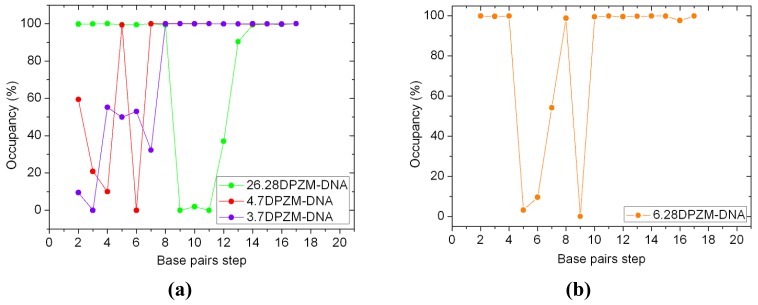

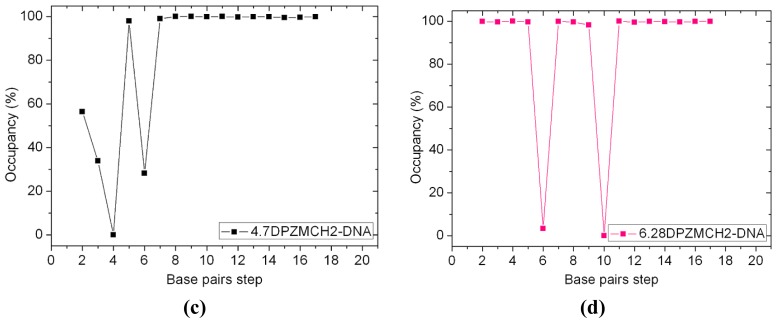
Hydrogen bond occupancies of the base pairs and the base-pair steps for the various platinum-DNA adducts (shown as circles for [Pt_2_(DPZM)Cl_2_(II)] and squares for [Pt_2_(DPZMCH2)Cl_2_(II)]). (**a**) 26.28DPZM-DNA (green), 4.7DPZM-DNA (red) and 3.7DPZM-DNA (violet); (**b**) 6.28DPZM-DNA (orange); (**c**) 4.7DPZMCH2-DNA (black); (**d**) 6.28 DPZMCH2-DNA (pink).

**Figure A4:**
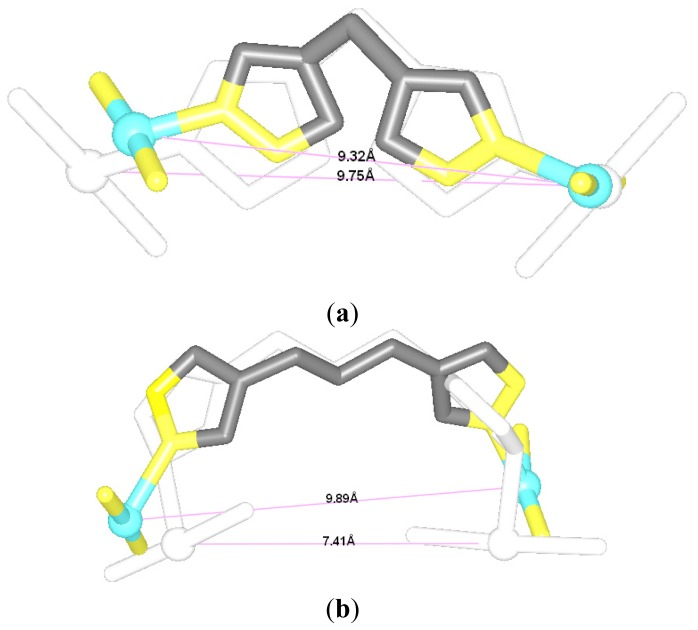
Overlap conformations between the crystal structures (white) and the averaged simulated structures of (**a**) [Pt_2_(DPZM)Cl_2_](II) (gray); and (**b**) [Pt_2_(DPZMCH2)Cl_2_](II) (gray). The platinum atoms are shown as white in the crystal structures, and light cyan in the averaged structures.

**Figure A5:**
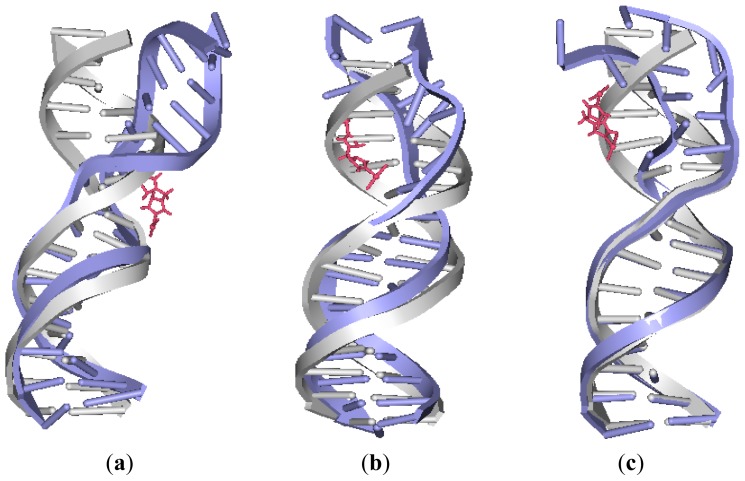
The averaged centroid structures of the platinum-DNA adduct and an undamaged B-DNA for: (**a**) 26.28DPZM-DNA; (**b**) 4.7DPZM-DNA; and (**c**) 3.7DPZM-DNA. [Pt_2_(DPZM)Cl_2_](II) is shown as pink, bound-DNA as blue, and B-DNA as gray.

**Figure A6:**
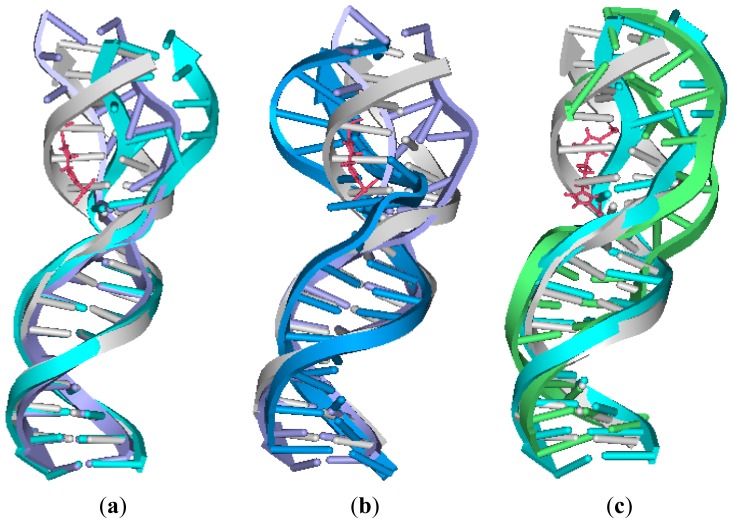
Overlap of the averaged structures for B-DNA (gray), [Pt_2_(DPZM)Cl_2_](II) and [Pt_2_(DPZMCH2)Cl_2_](II) (pink), and: (**a**) 4.7DPZM-DNA (blue) and 4.7DPZMCH2-DNA (cyan); (**b**) 4.7DPZM-DNA (blue) and 6.28DPZM-DNA (royal); and (**c**) 4.7DPZMCH2-DNA (cyan) and 6.28DPZMCH2-DNA (green).

**Chart A1:**
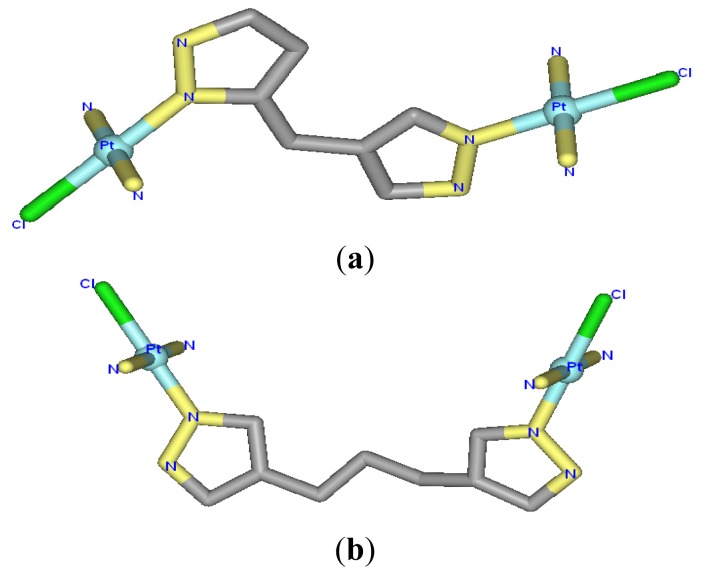
X-ray structures for (**a**) *trans*-[Pt_2_(DPZM)Cl_2_](II), and (**b**) *cis*-[Pt_2_(DPZMCH2)Cl_2_](II).

To confirm generalizability of these relationships in [Pt_2_(DTBPA)Cl_2_](II) and [Pt_2_(TPXA)Cl_2_](II) agents, the two additional dinuclear platinum agents, *trans*-[Pt_2_(DPZM)Cl_2_](II) (where DPZM = {(NH_3_)}_2_u-4,4′-dipyrazolylmethane) and *cis*-[Pt_2_(DPZMCH2)Cl_2_](II) (where DPZMCH2 = {(NH_3_)}_2_u-CH_2_CH_2_-4,4′-dipyrazolylmethane) binding to the same DNA molecule were simulated to study the characteristics of DNA distortion [42] (see Chart A1). In the [Pt_2_(DPZM)Cl_2_](II) agent, two *trans*-platinum centers are bridged by one acyclic alkane linker (–CH_2_–); each platinum center is coordinated by one chloride and three nitrogens in, respectively, ammonia, ammonia and pyrazole groups. To improve the flexibility of the [Pt_2_(DPZM)Cl_2_](II) agent, a relatively flexible linker (–CH_2_–CH_2_–CH_2_–) between two *cis*-platinum centers is introduced to the [Pt_2_(DPZMCH2)Cl_2_](II) agent. Six inter-/intra-strand cross-linking modes of 26.28DPZM-DNA, 4.7DPZM-DNA, 3.7DPZM-DNA, 6.28DPZM-DNA, 4.7DPZMCH2-DNA and 6.28DPZMCH2-DNA were built and simulated by using the same methods described in the “models and methods” section. The corresponding results for these six additional models were shown in Tables A1 and A2, Figures A2–A6. It can be seen from Tables A1 and A2, Figures A3 and A5 that the distortion of the average DNA conformation, compared with the B-DNA molecule, was larger for both 26.28DPZM-DNA and 3.7DPZM-DNA models than for the 4.7DPZM-DNA model due to the differences between the Pt–Pt distance of platinum agent and the N7–N7 distances of the B-DNA molecule. For example, the sum of the percentages of the hydrogen bonds destroyed was 400.06% for the 3.7DPZM-DNA model with this distance difference of 5.06 Å versus 319.72% for the 4.7DPZM-DNA model with that of 1.85 Å. The results further confirm that large differences between the platinum–platinum distance in the platinum agent and the platinum-bound N7–N7 distance in the DNA molecule can cause substantial distortions of the DNA conformation discussed above for the two [Pt_2_(DTBPA)Cl_2_](II) and [Pt_2_(TPXA)Cl_2_](II) agents. It can be seen from Table A1, Figures A3, A4 and A6 that the distortion of the DNA conformation for the rigid 4.7DPZM-DNA model with the deviation of DNA major groove width and depth of 9.65% and 30.06% was larger than that for the flexible 4.7DPZMCH2-DNA model with those of 5.08% and 13.35%, which confirms that the rigid platinum adduct may lead to more obvious distortion of the DNA backbone than the flexible platinum adduct. Moreover, for the *trans*-[Pt_2_(DPZM)Cl_2_](II) agent, the distortion of the DNA conformation (in comparison with the B-DNA molecule) was larger for the inter-strand cross-linking 6.28DPZM-DNA model than for the intra-strand cross-linking 4.7DPZM-DNA model; in contrast, for the *cis*-[Pt_2_(DPZMCH2)Cl_2_](II) agent, the DNA conformational distortion was larger for the intra-strand cross-linking 4.7DPZMCH2-DNA model than for the inter-strand cross-linking 6.28 DPZMCH2-DNA model, which further verifies that the *trans*-type platinum agents facilitate greater DNA conformational distortion through an inter-strand rather than an intra-strand cross-linking mode (see Table A1, Figures A3 and A6).

## 3. Discussions

### 3.1. Principal Component Analysis of the Major Conformational Dynamics

**Figure A7:**
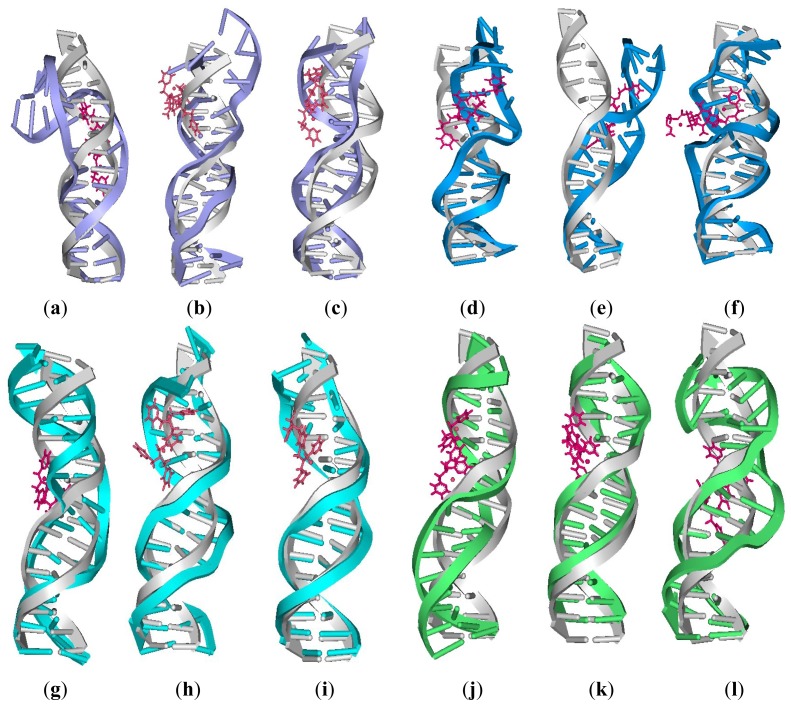
The average centroid structures of the 12 platinum-DNA adducts studied (pink for [Pt_2_(DTBPA)Cl_2_](II) and [Pt_2_(TPXA)Cl_2_](II); blue for intra-strand [Pt_2_(DTBPA)Cl_2_](II)-bound DNA; navy for inter-strand [Pt_2_(DTBPA)Cl_2_](II)-bound DNA; cyan for intra-strand [Pt_2_(TPXA)Cl_2_](II)-bound DNA, green for inter-strand [Pt_2_(TPXA)Cl_2_](II)-bound DNA; gray for B-DNA): (**a**) 26.28DTBPA-DNA; (**b**) 4.7DTBPA-DNA; (**c**) 3.7DTBPA-DNA; (**d**) 7.28DTBPA-DNA; (**e**) 6.28DTBPA-DNA; (**f**) 7.26DTBPA-DNA; (**g**) 26.28TPXA-DNA; (**h**) 4.7TPXA-DNA; (**i**) 3.7TPXA-DNA; (**j**) 7.28TPXA-DNA; (**k**) 6.28TPXA-DNA; (**l**) 7.26TPXA-DNA.

Principal component analysis was used to investigate the trajectories from the corresponding simulations to examine the dominant dynamic motions of the DNA in the 12 Pt_2_(DTBPA)/Pt_2_(TPXA)-DNA adducts studied. The first three principal components (PC1, PC2 and PC3) described about 90% of the essential modes of the dynamics for these adducts (Table 3). It was found that the first three components of the conformational motions roughly corresponded to a superposition of bending, unwinding and twisting motions. Visual analyses of averaged DNA structures from the trajectories support the dominant motions identified by PCA. The overall centroid structures for these adducts have been analyzed, and are shown in Figure A7. It may be seen that compared with the undamaged B-DNA conformation, there were obvious differences between the various cross-linking models in the DNA conformations of the platinum-DNA adducts (including bending, unwinding and twisting motions). In particular, the PC1 occupancy time percentages for binding of [Pt_2_(DTBPA)Cl_2_](II) to DNA were larger than those for [Pt_2_(TPXA)Cl_2_](II), which would be expected to lead to larger DNA bending angles for the rigid [Pt_2_(DTBPA)Cl_2_](II) agent than for the flexible [Pt_2_(TPXA)Cl_2_](II) agent (with respect to the undamaged B-DNA molecule) (see Table 3). The unwinding motions, in the vicinity of the platinum sites, for the Pt_2_(DTBPA)/Pt_2_(TPXA)-DNA adducts also efficiently induce DNA groove conformational changes; the first three motions take place simultaneously and are irreversible.

### 3.2. DNA Conformational Dynamics in the Platinum-DNA Adducts

**Figure 7:**
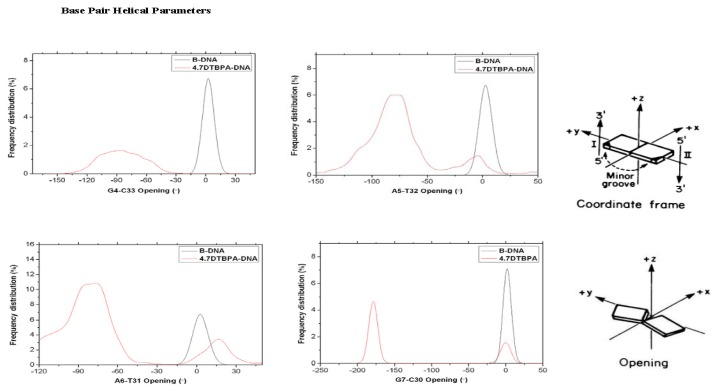

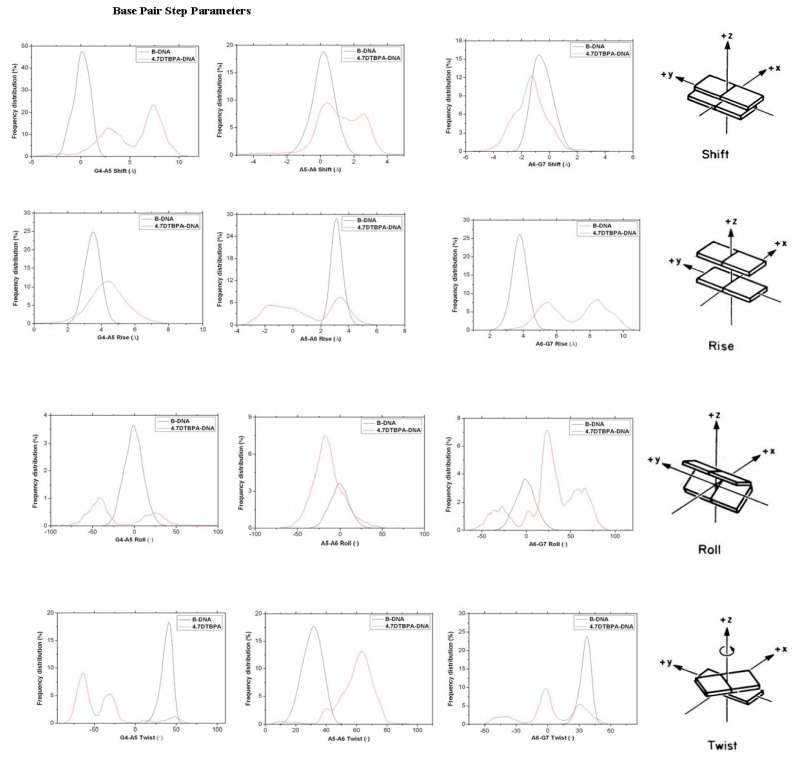
Selected frequency distributions of the representative DNA duplex base-pair helical/step parameters for the central binding base pairs of the 4.7DTBPA-DNA model (red line), compared with B-DNA (black line).

**Figure 8:**
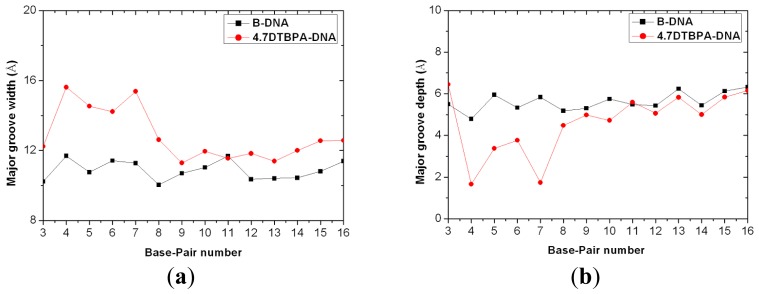
Major groove width (**a**) and depth (**b**) for the time-averaged structure of the DNA conformation of the 4.7DTBPA-DNA model (red line with circles), compared with B-DNA (black line with squares).

**Figure A8:**
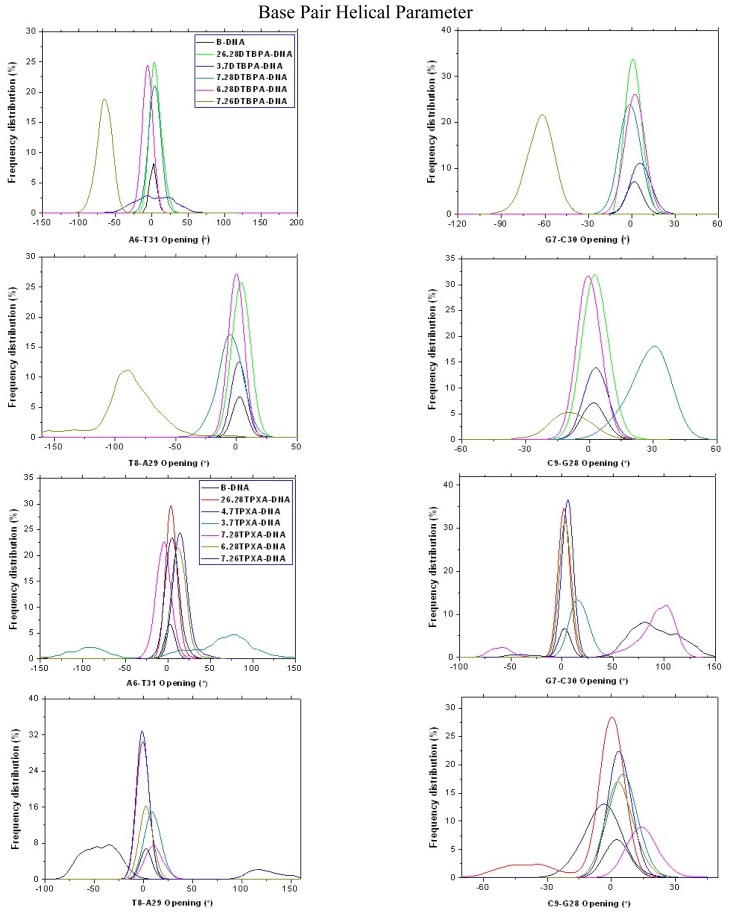

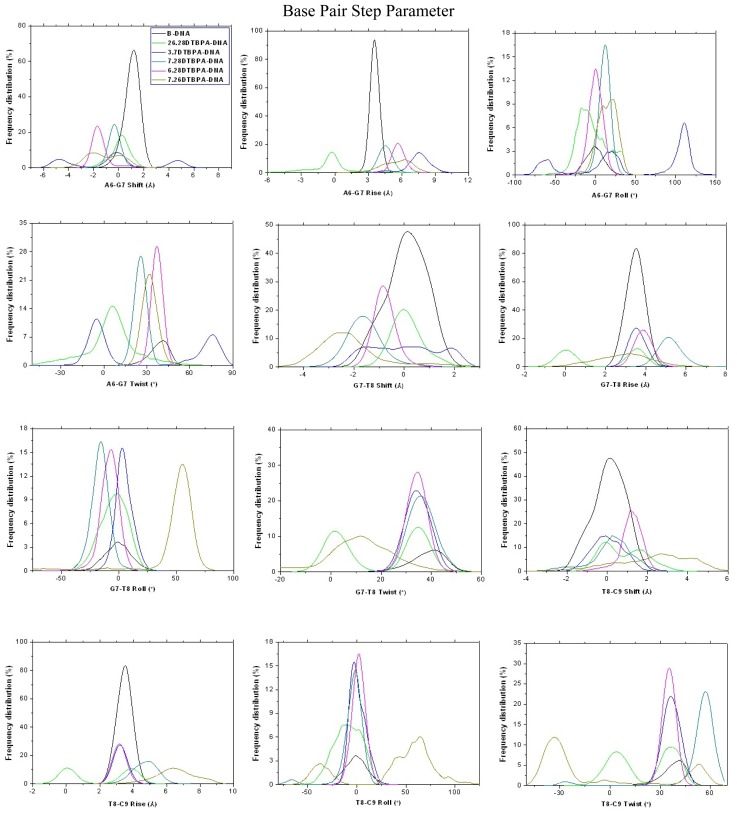
Selected frequency distributions of the representative DNA duplex base-pair helical parameters and base-pair step parameters for the central binding base-pairs of B-DNA (black), 26.28DTBPA-DNA (green), 3.7DTBPA-DNA (blue), 7.28DTBPA-DNA (dark cyan), 6.28DTBPA-DNA (magenta) and 7.26DTBPA-DNA (dark yellow); and 26.28TPXA-DNA (red), 4.7TPXA-DNA (blue), 3.7TPXA-DNA (dark cyan), 7.28TPXA-DNA (magenta), 6.28TPXA-DNA (dark yellow) and 7.26TPXA-DNA (navy).

**Figure A9:**
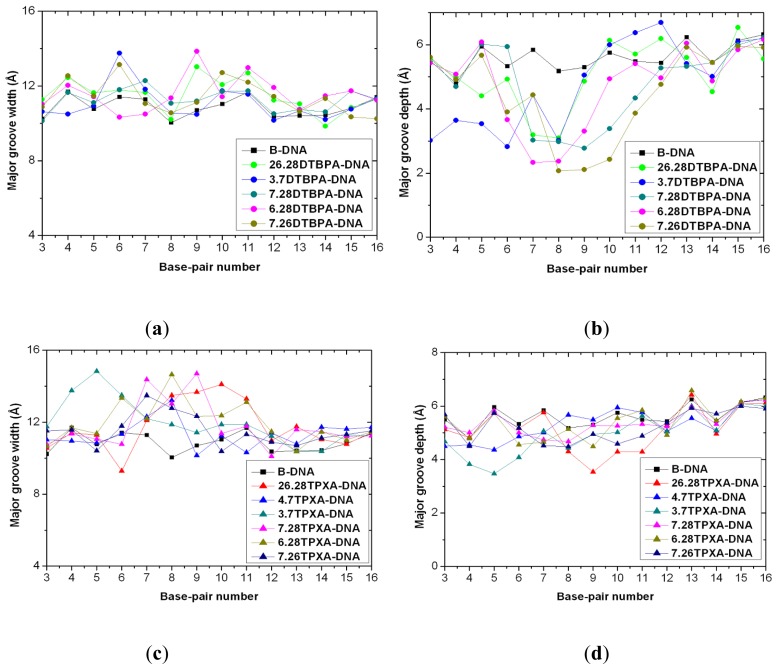
Major groove widths and depths for the time-averaged structures of the DNA conformations of the other 11 platinum-DNA adducts. Major groove width (**a**) and depth (**b**) for [Pt_2_(DTBPA)Cl_2_](II) are shown for: B-DNA (black squares); 26.28DTBPA-DNA (green circles); 3.7DTBPA-DNA (blue circles); 7.28DTBPA-DNA (dark cyan circles); 6.28DTBPA-DNA (magenta circles); and 7.26DTBPA-DNA (dark yellow circles). Major groove width (**c**) and depth (**d**) for [Pt_2_(TPXA)Cl_2_](II) are shown for: B-DNA (black squares); 26.28TPXA-DNA (red triangles); 4.7TPXA-DNA (blue triangles); 3.7TPXA-DNA (dark cyan triangles); 7.28TPXA-DNA (magenta triangles); 6.28TPXA-DNA (dark yellow triangles); and 7.26TPXA-DNA (navy triangles).

The [Pt_2_(DTBPA)Cl_2_](II) and [Pt_2_(TPXA)Cl_2_](II) agents disturb the DNA conformation through the covalent binding of the platinum centers to the N7 atoms of the DNA bases, and simultaneously, the locating of the corresponding large ligand of the agent to the major groove. The processes of DNA conformational dynamics result in changes in the DNA groove parameters. The frequency distributions of the DNA helical dynamics parameters have been analyzed, and are shown in Figure 7 for the 4.7DTBPA-DNA adduct, and in Figure A8 for the other 11 models of Pt_2_(DTBPA)/Pt_2_(TPXA)-DNA. In general, groove width and depth were measured mainly from the opening, twist and rise motions, and the shift motion, respectively, of the DNA base pair helixes or steps, as defined by CURVES analysis [43–45]. For example, for the 4.7DTBPA-DNA model (see Figure 7), the opening parameters of the DNA base pair helixes at connection sites G4:C33, A5:T32 and A6:T31 were changed ~−90°, ~−80° and ~−80°, respectively, away from the B-DNA molecule (compared with ~0° for the B-DNA molecule); the deviations in the twist angle values were ~20°, ~30° and ~−35° for the G4·A5, A5·A6 and A6·G7 base pair steps, with respect to the value of ~35° for the B-DNA molecule; and the rise value deviations were ~1.5 Å, ~−1 Å and ~5 Å for the G4·A5, A5·A6 and A6·G7 base pair steps, respectively, with respect to the value of ~3.5 Å for the B-DNA molecule. This results in a widening of the DNA major groove by 1.91 Å around base pairs G4:C33–G7:C30 (see Table 1 and Figure 8a), and the destruction of hydrogen bonds at base pairs G4:C33, A5:T32 and A6:T31 (see Figure 2a). The deviations in the shift values were ~8 Å, ~2 Å and ~−1 Å for the G4·A5, A5·A6 and A6·G7 base pair steps, respectively, compared with ~0 Å for the B-DNA molecule (see Figure 7); this results in a shoaling of the DNA major groove by 2.62 Å around base pairs G4:C33–G7:C30 (see Table 1 and Figure 8b). In addition, the DNA bend was measured by the twist, tilt and roll motions for DNA base pair steps. For example, for the 4.7DTBPA-DNA model (see Figure 7), the deviations in the values of the roll angles were ~−40°, ~−25°, and ~40° for the G4·A5, A5·A6 and A6·G7 base pair steps, with respect to ~0° for the B-DNA molecule; this contributes to a significant bending of the DNA helix toward the minor groove of 15.68°. Similar results were found for the other 11 models of Pt_2_(DTBPA)/Pt_2_(TPXA)-DNA (see Figure A9). Taken together, these data indicate that binding of the two types of platinum agent to the DNA molecule greatly affects the DNA groove parameters via dynamic changes in DNA conformation around the connected base pairs, leading to difficulties in DNA-repair as well as gene transcription.

## 4. Models and Methods

### 4.1. Starting Structures

The starting structures of all the platinum-DNA adducts contained an 18-mer duplex DNA sequence, 5′-d(GAAGAAGTCACAAAATGT)-3′, that was chosen on the basis of the breast/ovarian cancer-susceptibility gene, BRCA1 (Genbank #U14680) [10], and the [Pt_2_(DTBPA)Cl_2_](II) or [Pt_2_(TPXA)Cl_2_](II) agent selected from X-ray data [24,25]; see Chart 1. Previous studies have suggested that the two platinum center atoms may bind to the N7 atoms of the G/A bases of DNA, and might span 3–5 base pairs with intra-/inter-strand cross-links. Therefore, three types of intra-strand cross-link were constructed and simulated for [Pt_2_(DTBPA)Cl_2_](II), with 1232 atoms: an intra-1,3-GG cross-linking mode (designated 26.28DTBPA-DNA), an intra-1,4-GG cross-linking mode (designated 4.7DTBPA-DNA), and an intra-1,5-AG cross-linking mode (designated 3.7DTBPA-DNA); and three types of inter-strand cross-link were constructed and simulated for [Pt_2_(DTBPA)Cl_2_](II), with 1232 atoms: an inter-1,3-GG cross-linking mode (designated 7.28DTBPA-DNA), an inter-1,4-AG cross-linking mode (designated 6.28DTBPA-DNA), and an inter-1,5-GG cross-linking mode (designated 7.26DTBPA-DNA). The same protocol (with similar nomenclature) was applied to the [Pt_2_(TPXA)Cl_2_](II) agent. Three of these 12 adduct models, 26.28TPXA-DNA, 4.7TPXA-DNA and 7.28TPXA-DNA, have been previously examined using denaturing polyacrylamide gels and MALDI-TOF MS analysis [25]. The initial coordinates of the 12 platinum(II)-DNA adducts used in our simulations were generated by docking the platinum agent in the major groove of the DNA. For each binding adduct, several different orientations of the platinum compound, coordinating with the major groove of DNA, were chosen as the starting structures for our molecular dynamics simulations, thereby providing a good test of whether our molecular dynamics simulations were capable of driving significantly distinct starting structures to a non-distinguishable one when the simulations reached equilibrium. Bump-checking was turned on to ensure that no overlapping atoms were produced during the docking process. To compare the differences between the DNA adduct conformations and an undamaged DNA molecule, a bare-DNA (B-DNA) molecule simulation was also performed, in which an idealized B-DNA was used as a starting structure for the simulation. Given that each strand of DNA has numerous phosphate groups, 32 Na^+^ counterions were added to each system to achieve electroneutrality. The systems were explicitly solvated using the TIP3P water potential inside a box large enough to ensure that the solvent shell extended to 10 Å in all directions.

### 4.2. Force Field Parameter Preparation

The atoms for the studied platinum compound, excluding the platinum atoms themselves, were generated using the ANTECHAMBER module in the AMBER9 program [46]. The force field parameters and the RESP charges of the platinum compound were referenced from previous works [47–51]. Other force field parameters for the platinum compound were generated from the gaff force field in the AMBER9 program.

### 4.3. Molecular Dynamics Simulations and Trajectory Analysis

All molecular dynamics simulations were carried out using the AMBER9 package [46] with a classical AMBER parm99 [52,53] together with the parmbsc0 refinement [48,54] and gaff [55] force field parameters. Details of the molecular dynamics protocols are given in the Appendix.

### 4.4. Principal Component Analysis

Principal component analysis can be used to segregate large-scale correlated motions from random thermal fluctuations, thereby probing the essential dynamics of the system. Details of the analysis method are available in the Appendix.

### 4.5. DNA Groove Parameter Analyses of Trajectories

To investigate the distortion of the DNA, the frequency distributions (fraction of the time spent in each conformation) of the trajectories of the simulations for the models and a canonical B-DNA were calculated using the CURVES program [56]. To account for distortion of the whole DNA backbone, the overall bend, tilt and roll angles of the DNA time-averaged structures for the studied models were calculated from the CURVES outputs using MadBend [57]. Details of the calculation method are available in the Appendix.

## 5. Conclusions

Molecular dynamics simulations and DNA dynamics analyses for a series of [Pt_2_(DTBPA)Cl_2_](II)-DNA and [Pt_2_(TPXA)Cl_2_](II)-DNA adducts, with inter-/intra-strand cross-linking modes and 1–3, 1–4, 1–5 base intervals, were carried out to examine the distortions in the DNA double-helical structure. For each series of platinum agents, it was found that a larger difference between the platinum–platinum distance of the platinum agent and the platinum-bound N7–N7 distance of the DNA molecule was associated with a greater DNA conformational distortion. For the same cross-linking mode, the rigid [Pt_2_(DTBPA)Cl_2_](II) agent induced a larger DNA conformational distortion than the flexible [Pt_2_(TPXA)Cl_2_](II) agent. The *trans*-platinum configuration of [Pt_2_(DTBPA)Cl_2_](II) may facilitate conformational distortion of the platinum-DNA adduct through inter-strand cross-linking modes, whereas the *cis*-platinum configuration of [Pt_2_(TPXA)Cl_2_](II) may favor intra-strand cross-linking modes; this is consistent with previous studies [50–52]. DNA dynamics analysis revealed that the DNA base motions (opening, shifting, rising, rolling and twisting away from the DNA grooves) caused by the binding of the platinum agent induced widening of the DNA major and minor grooves and destruction of the hydrogen bonds of the DNA base pairs. These observations are validated for the other two *trans*-[Pt_2_(DPZM)Cl_2_](II) and *cis*-[Pt_2_(DPZMCH2)Cl_2_](II) agents binding to DNA. Our calculated results provide useful insights, in atomic detail, into how DNA conformations are affected by platinum agents containing different ligands and linkers, and may aid the experimental design of novel types of platinum-based anticancer agent.

## Figures and Tables

**Table 1:** The widths and depths of the minor and major grooves and the bend angles for the time-averaged structures of the DNA conformations for the Pt_2_(DTBPA)/Pt_2_(TPXA)-DNA adducts and B-DNA.

Model	Major groove width (Å)	Major groove depth (Å)	Minor groove width (Å)	Minor groove depth (Å)	Bend (°)
26.28DTBPA-DNA	12.47	3.28	5.65	4.93	35.48
4.7DTBPA-DNA	13.72	3.22	5.95	5.03	30.70
3.7DTBPA-DNA	13.09	4.94	6.76	4.15	48.62
7.28DTBPA-DNA	14.85	2.59	6.21	5.08	53.81
6.28DTBPA-DNA	15.76	2.46	6.12	4.29	49.91
7.26DTBPA-DNA	13.66	4.62	5.03	4.85	61.94
26.28TPXA-DNA	12.27	5.70	5.91	5.04	27.20
4.7TPXA-DNA	12.43	7.01	6.81	3.71	26.18
3.7TPXA-DNA	10.52	6.18	6.53	3.27	49.15
7.28TPXA-DNA	12.95	6.89	5.52	3.83	30.58
6.28TPXA-DNA	11.98	5.73	6.04	4.28	27.15
7.26TPXA-DNA	9.73	6.40	6.35	3.97	50.57
B-DNA	11.81	5.84	5.87	4.64	15.02

**Table 2:** Differences between the platinum-binding atoms of the base pairs (N7–N7) of bare DNA and the Pt_2_(DTBPA)-DNA adduct, before and after simulation (Δ(Pt–N7)); and between the Pt–Pt distance in the [Pt_2_(DTBPA)Cl_2_](II) agent and N7–N7 distance in bare DNA (Δ(N7–N7)).

Model	Pt–Pt distance (Å)	N7–N7 distance		Δ(Pt–N7) (Å)	Δ(N7–N7) (Å)

		B-DNA (Å)	Platinum-DNA adduct (Å)		
3.7DTBPA-DNA	9.70	14.81	11.05	5.11	3.76
4.7DTBPA-DNA	9.70	11.60	11.22	1.90	0.38
26.28DTBPA-DNA	9.70	7.63	9.85	2.07	2.22

**Table 3:** Occupancy time percentages for the first three principal components during the simulations for each of the studied Pt_2_(DTBPA)/Pt_2_(TPXA)-DNA adducts (PCsum represents the sum of PC1, PC2 and PC3).

Model	PC1	PC2	PC3	PCsum
26.28DTBPA-DNA	77.60	7.74	7.07	92.41
4.7DTBPA-DNA	66.56	22.98	1.98	91.52
3.7DTBPA-DNA	48.93	32.79	12.50	94.22
7.28DTBPA-DNA	67.36	20.31	5.89	93.56
6.28DTBPA-DNA	67.37	22.45	3.12	92.94
7.26DTBPA-DNA	54.53	21.73	16.58	92.84
26.28TPXA-DNA	60.40	26.49	4.22	91.11
4.7TPXA-DNA	58.00	24.51	7.66	90.17
3.7TPXA-DNA	63.53	19.50	8.80	91.83
7.28TPXA-DNA	62.46	19.07	8.45	89.98
6.28TPXA-DNA	40.14	331.45	116.72	888.31
7.26TPXA-DNA	50.27	24.79	15.20	90.26

**Table A1:** The widths and depths of the minor and major grooves and the bend angles for the time-averaged structures of the DNA conformations for the various Pt_2_(DPZM)/Pt_2_(DPZMCH2)-DNA adducts and B-DNA.

Model	Major groove width (Å)	Major groove depth (Å)	Minor groove width (Å)	Minor groove depth (Å)	Bend(°)
26.28DPZM-DNA	13.27	6.51	4.67	5.64	43.91
4.7DPZM-DNA	12.95	4.49	6.42	4.14	30.88
3.7DPZM-DNA	13.11	4.35	6.23	4.36	34.94
6.28DPZM-DNA	15.40	2.51	4.16	3.79	48.73
4.7DPZMCH2-DNA	12.41	6.62	6.04	3.98	21.92
6.28DPZMCH2-DNA	11.47	6.23	6.51	4.16	19.24
B-DNA	11.81	5.84	5.87	4.64	15.02

**Table A2:** Differences between the platinum-binding atoms of the base pairs (N7–N7) of bare DNA and the Pt_2_(DPZM)-DNA adduct, before and after simulation (Δ(Pt–N7)); and between the Pt–Pt distance in the Pt_2_(DPZM)Cl_2_ agent and N7–N7 distance in bare DNA (Δ(N7–N7)).

Model	Pt–Pt distance (Å)	N7–N7 distance		Δ(Pt–N7) (Å)	Δ(N7–N7) (Å)

		B-DNA (Å)	Platinum-DNA adduct (Å)		
3.7DPZM-DNA	9.75	14.81	12.80	5.06	2.01
4.7DPZM-DNA	9.75	11.60	11.47	1.85	0.13
26.28DPZM-DNA	9.75	7.63	11.07	2.12	3.44

## Appendix

### Molecular Dynamics Simulation Protocols

All molecular dynamics (MD) simulations were carried out using the AMBER9 package [37,58] with a classical AMBER parm99 [43,44] together with the parmbsc0 refinement [39,45] and gaff [46] force field parameters. The protocol for all MD simulations was as follows: (1) All systems were submitted to energy minimization to remove unfavorable contacts. Four cycles of minimizations were performed with 2500 steps in each minimization, with harmonic restraints ranging from 100 kcal mol^−1^Å^−2^, 25 kcal mol^−1^Å^−2^ to 10 kcal mol^−1^Å^−2^ on the DNA and platinum compound positions. Finally, 5000 steps of unrestrained minimization were carried out before the heating process. The cutoff distance used for non-bonded interactions was 10 Å. The SHAKE algorithm [59] was used to restrain the bonds containing hydrogen. (2) Each energy-minimized structure was heated over 120 ps from 0 to 300 K (with a temperature coupling of 0.2 ps) in a constant volume, while the positions of DNA and the platinum compound were restrained with a small value of 10 kcal mol^−1^Å^−2^. A constant volume was maintained during the processes. (3) An unrestrained equilibration was carried out for 200 ps under constant pressure and constant temperature conditions, before a trajectory was generated for a further simulation. The temperature and pressure were allowed to fluctuate at around 300 K and 1 bar, respectively, with a corresponding coupling of 0.2 ps. For each simulation, an integration step of 2 fs was used. (4) Production runs of 50 ns were carried out using the same protocol. In each simulation, the time point at 200 ps after thermal equilibration was selected as the starting point for data collection. During the production run, 25,000 structures from each simulation were saved for post-processing by uniformly sampling the trajectory. (5) Finally, simulated annealing was performed for each system by allowing the system to cool from 300 K to 0 K during the course of a 1-ns simulation. The conformational free energies for all systems were calculated, using MM-PBSA energy analysis, from 1000 snapshots extracted at 5-ps intervals during the last 10 ns of each simulation. The MM-PBSA free energy computations were carried out using the AMBER9 program.

### Principal Component Analysis

Principal component analysis (PCA) was used to identify the dominant motions observed during a simulation by visual inspection; this was achieved by calculating the eigenvectors from the covariance matrix of a simulation and then filtering the trajectories along each of the different eigenvectors. As PCA reduces the dimensionality of the trajectory data, by using only the first several principal components we can extract important dynamical features describing the collective and overall motions of the system [60–63]. The platinum-DNA adduct systems reached equilibrium after 10 ns; thus, the final 40 ns of the equilibrated MD simulations of the 20,000 trajectories for each of the platinum-DNA adducts was subjected to PCA analysis. The first three principal components described >90% of the essential modes of the dynamics for the platinum-adducted DNA complexes. The dynamics of the first three principal components were visualized using the Discovery Studio molecular graphics tool.

### DNA Groove Parameter Analyses of the Trajectories

The PTRAJ module of the AMBER9 program was used to extract the produced conformations. These extracted snapshots were saved in the Protein Data Bank (PDB) format. Each nucleotide type was converted from the AMBER format to PDB format, and the resulting snapshots were submitted to the CURVES program [47]. The following CURVES parameters were extracted: global base-base parameters (shear, stretch, stagger, buckle, propeller and opening); and global inter-base-pair parameters (shift, slide, rise, tilt, roll and twist). Percentage occupancy distributions of the DNA helical parameters were calculated by normalizing the frequency distributions to 100%.

The overall bend, tilt and roll angles of the DNA for the averaged structures of these adducts were calculated from the CURVES output using MadBend (Department of Chemistry and Courant Institute of Mathematical Sciences, New York University and Howard Hughes Medical Institute, New York, NY, USA http://www.biomath.nyu.edu/index/software/Madbend/index.html), developed by Strahs and Schlick [48]. This method evaluates the DNA curvature by summing the projected components of local base-pair step tilt and roll angles after adjusting the helical twist. Bends in the helical axis defined by a negative roll angle indicate bending toward the minor groove, while bends defined by a positive roll angle correspond to bending toward the major groove [48].

## References

[1] Wang D., Lippard S.J. (2005). Cellular processing of platinum anticancer drugs. Nat. Rev. Drug Discov.

[2] Jung Y., Lippard S.J. (2007). Direct cellular responses to platinum-induced DNA damage. Chem. Rev.

[3] Steer C.B., Chrystal K., Cheong K.A., Galani E., Marx G.M., Strickland A.H., Yip D., Lofts F., Gallagher C., Thomas H. (2006). Gemcitabine and oxaliplatin followed by paclitaxel and carboplatin as first line therapy for patients with suboptimally debulked, advanced epithelial ovarian cancer. A phase II trial of sequential doublets. The GO-First Study. Gynecol. Oncol.

[4] McWhinney S.R., Goldberg R.M., McLeod H.L. (2009). Platinum neurotoxicity pharmacogenetics. Mol. Cancer Ther.

[5] Kormos B.L., Benitex Y., Baranger A.M., Beveridge D.L. (2007). Affinity and specificity of protein U1A-RNA complex formation based on an additive component free energy model. J. Mol. Biol.

[6] Reedijk J (2003). New clues for platinum antitumor chemistry: Kinetically controlled metal binding to DNA. Proc. Natl. Acad. Sci. USA.

[7] Park G.Y., Wilson J.J., Song Y., Lippard S.J. (2012). Phenanthriplatin, a monofunctional DNA-binding platinum anticancer drug candidate with unusual potency and cellular activity profile. Proc. Natl. Acad. Sci. USA.

[8] Pizarro A.M., Sadler P.J. (2009). Unusual DNA binding modes for metal anticancer complexes. Biochimie.

[9] Silverman A.P., Bu W., Cohen S.M., Lippard S.J. (2002). 2.4 Å Crystal structure of the asymmetric platinum complex {Pt (ammine)(cyclohexylamine)}^2+^ bound to a dodecamer DNA duplex. J. Biol. Chem.

[10] Zhu Y., Wang Y., Chen G (2009). Differences in conformational dynamics of [Pt_3_ (HPTAB)]^6+^-DNA adducts with various cross-linking modes. Nucleic Acids Res.

[11] Kasparkova J., Mackay F.S., Brabec V., Sadler P.J. (2003). Formation of platinated GG cross-links on DNA by photoactivation of a platinum(IV) azide complex. J. Biol. Inorg. Chem.

[12] Klein A.V., Hambley T.W. (2009). Platinum drug distribution in cancer cells and tumors. Chem. Rev.

[13] Agarwal R., Kaye S.B. (2003). Ovarian cancer: Strategies for overcoming resistance to chemotherapy. Nat. Rev. Cancer.

[14] Ramachandran S., Temple B.R., Chaney S.G., Dokholyan N.V. (2009). Structural basis for the sequence-dependent effects of platinum-DNA adducts. Nucleic Acids Res.

[15] Shah N., Dizon D.S. (2009). New-generation platinum agents for solid tumors. Future Oncol.

[16] Kelland L (2007). Broadening the clinical use of platinum drug-based chemotherapy with new analogues. Expert Opin. Invest. Drugs.

[17] Hegmans A., Berners-Price S.J., Davies M.S., Thomas D.S., Humphreys A.S., Farrell N (2004). Long range 1,4 and 1,6-interstrand cross-links formed by a trinuclear platinum complex. Minor groove preassociation affects kinetics and mechanism of cross-link formation as well as adduct structure. J. Am. Chem. Soc.

[18] Mambanda A., Jaganyi D., Hochreuther S., van Eldik R (2010). Tuning the reactivity of chelated dinuclear Pt(II) complexes through a flexible diamine linker. A detailed kinetic and mechanistic study. Dalton Trans.

[19] Zerzankova L., Kostrhunova H., Vojtiskova M., Novakova O., Suchankova T., Lin M.X., Guo Z.J., Kasparkova J., Brabec V (2010). Mechanistic insights into antitumor effects of new dinuclear *cis* Pt(II) complexes containing aromatic linkers. Biochem. Pharmacol.

[20] Ruhayel R.A., Zgani I., Berners-Price S.J., Farrell N.P. (2011). Solution studies of dinuclear polyamine-linked platinum-based antitumour complexes. Dalton Trans.

[21] Kida N., Katsuda Y., Yoshikawa Y., Komeda S., Sato T., Saito Y., Chikuma M., Suzuki M., Imanaka T., Yoshikawa K (2010). Characteristic effect of an anticancer dinuclear platinum(II) complex on the higher-order structure of DNA. J. Biol. Inorg. Chem.

[22] Zhu J.H., Lin M.X., Fan D.M., Wu Z.Y., Chen Y.C., Zhang J.F., Lu Y., Guo Z.J. (2009). The role of bridging ligands in determining DNA-binding ability and cross-linking patterns of dinuclear platinum(II) antitumour complexes. Dalton Trans.

[23] Farrell N (2004). Polynuclear platinum drugs. Met. Ions Biol. Syst.

[24] Xu Z., Zhang Y., Xue Z., Yang X., Wu Z., Guo Z (2009). DNA-binding property and antitumor activity of a cyclam bridged dinuclear platinum (II) complex. Inorg. Chim. Acta.

[25] Zhu J., Zhao Y., Zhu Y., Wu Z., Lin M., He W., Wang Y., Chen G., Dong L., Zhang J. (2009). DNA cross-linking patterns induced by an antitumor-active trinuclear platinum complex and comparison with its dinuclear analogue. Chem.Eur. J.

[26] Malina J., Kasparkova J., Farrell N.P., Brabec V (2011). Walking of antitumor bifunctional trinuclear PtII complex on double-helical DNA. Nucleic Acids Res.

[27] Sharma S., Gong P., Temple B., Bhattacharyya D., Dokholyan N.V., Chaney S.G. (2007). Molecular dynamic simulations of cisplatin-and oxaliplatin-d (GG) intrastand cross-links reveal differences in their conformational dynamics. J. Mol. Biol.

[28] Arriagada R., Bergman B., Dunant A., le Chevalier T., Pignon J.P., Vansteenkiste J (2004). Cisplatin-based adjuvant chemotherapy in patients with completely resected non-small-cell lung cancer. N. Engl. J. Med.

[29] Harris A.L., Yang X., Hegmans A., Povirk L., Ryan J.J., Kelland L., Farrell N.P. (2005). Synthesis, characterization, and cytotoxicity of a novel highly charged trinuclear platinum compound. Enhancement of cellular uptake with charge. Inorg. Chem.

[30] Kapp T., Muller S., Gust R (2006). Dinuclear alkylamine platinum (II) complexes of [1,2-bis (4-fluorophenyl) ethylenediamine] platinum (II): Influence of endocytosis and copper and organic cation transport systems on cellular uptake. ChemMedChem.

[31] Mangrum J.B., Farrell N.P. (2010). Excursions in polynuclear platinum DNA binding. Chem. Commun.

[32] Montero E.I., Benedetti B.T., Mangrum J.B., Oehlsen M.J., Qu Y., Farrell N.P. (2007). Pre-association of polynuclear platinum anticancer agents on a protein, human serum albumin. Implications for drug design. Dalton Trans.

[33] Washizu H., Kikuchi K (2006). Electric polarizability of DNA in aqueous salt solution. J. Phys. Chem. B.

[34] Bao G., Suresh S (2003). Cell and molecular mechanics of biological materials. Nat. Mater.

[35] Komeiji Y., Mochizuki Y., Nakano T., Mori H, Wang L.C. (2012). Recent advances in fragment molecular orbitalbased molecular dynamics (FMO-MD) simulations. Molecular Dynamics-Theoretical Developments and Applications in Nanotechnology and Energy.

[36] Mori H., Hirayama N., Komeiji Y., Mochizuki Y (2012). Differences in hydration between *cis*- and *trans-* platin: Quantum insights by *ab initio* fragment molecular orbital-based molecular dynamics (FMO-MD). Comput. Theor. Chem.

[37] Srinivasan J., Cheatham T.E., Cieplak P., Kollman P.A., Case D.A. (1998). Continuum solvent studies of the stability of DNA, RNA, and phosphoramidate-DNA helices. J. Am. Chem. Soc.

[38] Yang D., van Boom S.S.G.E., Reedijk J., van Boom J.H., Wang A.H.J. (1995). Structure and isomerization of an intrastrand cisplatin-cross-linked octamer DNA duplex by NMR analysis. Biochemistry.

[39] Spingler B., Whittington D.A., Lippard S.J. (2001). 2.4 Å crystal structure of an oxaliplatin1,2-d (GpG) intrastrand cross-link in a DNA dodecamer duplex. Inorg. Chem.

[40] Marzilli L.G., Saad J.S., Kuklenyik Z., Keating K.A., Xu Y (2001). Relationship of solution and protein-bound structures of DNA duplexes with the major intrastrand cross-link lesions formed on cisplatin binding to DNA. J. Am. Chem. Soc.

[41] Wheate N.J., Collins J.G. (2003). Multi-nuclear platinum complexes as anti-cancer drugs. Coordin. Chem. Rev.

[42] Grant Collins J., Wheate N.J. (2004). Potential adenine and minor groove binding platinum complexes. J. Inorg. Biochem.

[43] Stofer E., Lavery R (1994). Measuring the geometry of DNA grooves. Biopolymers.

[44] Poncin M., Piazzola D., Lavery R (1992). DNA flexibility as a function of allomorphic conformation and of base sequence. Biopolymers.

[45] Goodsell D.S., Dickerson R.E. (1994). Bending and curvature calculations in B-DNA. Nucleic Acids Res.

[46] Case D.A., Darden T.A., Cheatham I.T.E., Simmerling C.L., Wang J.M., Duke R.E., Luo R., Merz K.M., Pearlman D.A., Crowley M (2006). AMBER9.

[47] Pople J., Nesbet R (1954). Self-consistent orbitals for radicals. J. Chem. Phys.

[48] Yao S., Plastaras J.P., Marzilli L.G. (1994). A molecular mechanics AMBER-type force field for modeling platinum complexes of guanine derivatives. Inorg. Chem.

[49] McWeeny R., Diercksen G. (1968). Self-consistent perturbation Theory. II. Extension to open shells. J. Chem. Phys.

[50] Chval Z., Sip M (1998). Force field for platinum binding to adenine and guanine taking into account flexibility of nucleic acids bases. J. Phys. Chem. B.

[51] Cundari T.R., Fu W., Moody E.W., Slavin L.L., Snyder L.A., Sommerer S.O., Klinckman T.R. (1996). Molecular mechanics force field for platinum coordination complexes. J. Phys. Chem.

[52] Duan Y., Wu C., Chowdhury S., Lee M.C., Xiong G., Zhang W., Yang R., Cieplak P., Luo R., Lee T (2003). A point-charge force field for molecular mechanics simulations of proteins based on condensed-phase quantum mechanical calculations. J. Comput. Chem.

[53] Lee M.C., Duan Y (2004). Distinguish protein decoys by using a scoring function based on a new AMBER force field, short molecular dynamics simulations, and the generalized born solvent model. Proteins Struct. Funct. Bioinf.

[54] Perez A., Marchan I., Svozil D., Sponer J., Cheatham T.E., Laughton C.A., Orozco M (2007). Refinement of the AMBER force field for nucleic acids: Improving the description of [alpha]/[gamma] conformers. Biophys. J.

[55] Wang J., Wolf R.M., Caldwell J.W., Kollman P.A., Case D.A. (2004). Development and testing of a general amber force field. J. Comput. Chem.

[56] Lavery R., Sklenar H (1988). The definition of generalized helicoidal parameters and of axis curvature for irregular nucleic acids. J. Biomol. Struct. Dyn.

[57] Strahs D., Schlick T (2000). A-tract bending: Insights into experimental structures by computational models. J. Mol. Biol.

[58] Pendyala L., Creaven P (1993). *In vitro* cytotoxicity, protein binding, red blood cell partitioning, and biotransformation of oxaliplatin. Cancer Res.

[59] Miyamoto S., Kollman P.A. (1992). Settle: An analytical version of the SHAKE and RATTLE algorithm for rigid water models. J. Comput. Chem.

[60] Amadei A., Linssen A., Berendsen H.J.C. (1993). Essential dynamics of proteins. Proteins Struct. Funct. Bioinf.

[61] Yamaguchi H., van Aalten D.M.F., Pinak M., Furukawa A., Osman R (1998). Essential dynamics of DNA containing a cis.syn cyclobutane thymine dimer lesion. Nucleic Acids Res.

[62] Teeter M.M., Case D.A. (1990). Harmonic and quasiharmonic descriptions of crambin. J. Phys. Chem.

[63] Van Aalten D., Findlay J., Amadei A., Berendsen H (1995). Essential dynamics of the cellular retinol-binding protein—Evidence for ligand-induced conformational changes. Protein Eng.

